# Migrant health research in the Republic of Ireland: a scoping review

**DOI:** 10.1186/s12889-019-6651-2

**Published:** 2019-03-20

**Authors:** Nazmy Villarroel, Ailish Hannigan, Santino Severoni, Soorej Puthoopparambil, Anne MacFarlane

**Affiliations:** 1 The Graduate Entry Medical School, University Of Limerick Campus, Plassey Park Road, Castletroy Co., Limerick, V94T9PX Ireland; 2 Health Research Institute, University of Limerick, Limerick, Ireland; 3 Migration and Health programme, Division of Policy and Governance for Health and Well-being, WHO Regional Office for Europe, København, Denmark; 4 International Maternal and Child Health (IMCH), Department of Women’s and Children’s Health, Uppsala University, Uppsala, Sweden

**Keywords:** Immigrant, Health, Ireland, Scoping review, World health organization

## Abstract

**Background:**

Migration to European countries has increased in number and diversity in recent years. Factors such as access to healthcare, language barriers and legal status can impact the health outcomes of migrant groups. However, little is known about the evidence base on the health status of migrants in the Republic of Ireland. Our aim was to scope existing peer-reviewed research on the health of migrants in Ireland and identify any gaps in the evidence.

**Methods:**

We conducted a scoping review of peer-reviewed research on the health of migrants in the Republic of Ireland. Eleven electronic databases were searched for peer-reviewed, empirical articles published between 2001 and 2017. Search terms were adapted from a World Health Organisation review. Findings were analysed using the 2016 World Health Organisation Strategy and Action Plan for Refugee and Migrant Health in the World Health Organisation European region, which outlines nine strategic areas that require collaborative action.

**Results:**

Of 9396 articles retrieved, 80 met inclusion criteria, with the majority (81%) published since 2009. More than half of the studies had a quantitative design (65%). Migrants studied came from Eastern Europe, Asia and Africa and included labour migrants, refugees and asylum seekers. Most studies related to two World Health Organisation strategic areas; 4: “achieving public health preparedness and ensuring an effective response”, and 5: “strengthening health systems and their resilience”.

**Conclusion:**

There is growing attention to migrant health in Ireland with a balance of qualitative and quantitative research. While much of the identified research is relevant to three of the World Health Organisation strategic areas, there are significant gaps in the other six areas. The study design could be replicated in other countries to examine and inform migrant health research.

**Electronic supplementary material:**

The online version of this article (10.1186/s12889-019-6651-2) contains supplementary material, which is available to authorized users.

## Background

Migration is a longstanding and global phenomenon. People migrate for many reasons and there is no universally accepted definition of ‘migrant’. This has consequences for public health because it can impact on eligibility for healthcare and it presents difficulties in developing a standardised evidence base [1]. For the purpose of this paper, we employ a broad definition of ‘migrant’ from the International Organization for Migration (IOM), which is also used in World Health Assembly (WHA) resolutions 61.17 and 70.15 (see Table 1) [2, 3]. References to refugees are made following the definition from United Nations High Commissioner for Refugees (UNHCR) (see Table 1) [4].

**Table 1:** Definitions of ‘migrant’ and ‘refugee’

Migrant	Any person who is moving or has moved across an international border or within a State away from his/her habitual place of residence, regardless of the person’s legal status, whether the movement is voluntary or involuntary, what the causes for the movement are and what the length of the stay is. (IOM)
Refugee	Someone who has been forced to flee his or her country because of persecution, war or violence. A refugee has a well-founded fear of persecution for reasons of race, religion, nationality, political opinion or membership in a particular social group. Most likely, they cannot return home or are afraid to do so. (UNHCR)

Migration has become an increasingly significant phenomenon in Europe. Based on the most recent report on world migration from the IOM, 4.7 million people migrated to one of the European Union (EU)-28 countries in 2015, compared with approximately 1.8 million people in 2005 [5, 6]. Consequently, a number of European countries with long histories of emigration have experienced unprecedented inward patterns of migration, including Spain, Portugal and the Republic of Ireland [7]. The focus in this paper is on the Irish setting.

There are multiple types of migration to Ireland, [8] EU/European Economic Area (EEA) nationals who are free to move, live and work in Ireland with no special permission; migrants who move for work or study reasons, through marriage, civil partnership or close family relationship; and the asylum system. In addition, there are Refugee Protection Programmes (RPP) established by the Government.

Migration patterns in the Republic of Ireland began to change in a significant way from the late 1990s. There was a sharp rise in the number of asylum applications in 1999, from several hundred to over 7000 per annum; this peaked at just over 10,000 in 2000 and 2001 [9, 10]. Subsequently, the number of labour migrants (defined as those seeking work or employed in the host country), [11] also rose during an economic boom in Ireland and the enlargement of the EU during the 2000s [7]. In late 2008, the country encountered a severe economic recession. As a result, there was increased *outward* migration and decreased *inward* migration. [8] explains that the latter was related to three factors: a drop in asylum applications across Europe, the impacts of EU enlargement in 2004 on intra-EU migration patterns, and the rise in unemployment as a consequence of the recession in Ireland. The number of immigrants coming to Ireland rose again by 2014 [12].

According to the latest Census (2016), net inward migration has increased significantly [13]. Current figures show that 17% of the population were born abroad; [13] the fifth highest rate in the EU-28. Ireland has the ninth highest proportion of migrants in the World Health Organisation (WHO) European region [14, 15]. At the last Census, the top five countries of origin for non-Irish nationals in Ireland are Poland, the United Kingdom, Lithuania, Romania and Latvia [13].

The census data shows that the gender balance of non-Irish nationals is almost evenly split. The age profile is younger compared with the general population: Nearly half of all non-Irish nationals are aged between 25 and 42 compared with less than a quarter of Irish nationals [13]. Patterns of family and household arrangements differ across groups. For example. Spanish, Brazilian, Italian and French nationals were most likely to be single while Indian nationals had the largest proportion of married persons [13]. Almost 15% of the workforce is Ireland is made up of non-Irish nationals with the largest numbers working in wholesale and retail sectors. The unemployment rate for non-Irish nationals is nearly 3% higher than Irish (15.4% compared with 12.5%) and there was a relationship between higher educational status and employment status [13].

In 2017, there were 1910 new asylum applications (the five top countries of origin were Syria, Georgia, Albania, Zimbabwe and Pakistan) and 5670 pending applications (the five top countries of origin were Pakistan, Albania, Zimbabwe, Nigeria, Georgia [16]. In addition, the most recent RPP was established by Government Decision in 2015 as a direct response to the humanitarian crisis that developed in Southern Europe as a consequence of mass migration from areas of conflict in the Middle East and Africa [17]. This has three strands:

- Relocation Strand for asylum seekers from Greece and Italy through the EU relocation mechanism established by two EU Council Decisions in 2015
- Resettlement Strand for programme refugees focused on resettling UNHCR refugees from Lebanon
- Other mechanisms such as the acceptance of unaccompanied minors previously resident in the unofficial camp in Calais, France [17].

One of the great challenges of this newly diverse population for the Irish State is to adequately support the health of migrants. Although mortality rates in migrants are sometimes below those of the host population, [18, 19] available data suggest that they tend to be more vulnerable to certain communicable diseases, occupational health hazards, injuries, poor mental health, diabetes mellitus, and maternal and child health problems [20]. In addition, the health of refugees on the move in Europe is jeopardised by their poor living circumstances and barriers to accessing healthcare [21]. Once in the host country, refugees and migrants can encounter barriers to healthcare services in terms of their entitlement to healthcare, communication and language difficulties and attitudinal discrimination [22]. Previous studies investigating the health of migrants compared with host populations in Europe have shown mixed results. A review on the health status of migrants in Europe showed that the migrant population appeared to be more disadvantaged compared to the host population [23]. Another study found that most countries had either higher or lower health service utilisation patterns among migrants, depending on the type of health service used, e.g. utilisation of emergency services is higher, while utilisation of specialist services is lower [24].

It is important to strengthen the evidence base in Ireland on migrant health in order to develop knowledge about any such differences between migrants and the Irish-born population. A review of Irish research on refugees’ and asylum seekers’ health was carried out in 2001 and again in 2004 [25, 26]. However, there has been no review since. This is problematic for several reasons. First, there is a sustained increase of inward migration, including through the aforementioned Government’s recent involvement in the Refugee Resettlement Programme [17]. Second, there is an emerging cohort of children of migrants living in Ireland for the first time in Irish history. Third, there have been a series of initiatives and developments in policy and service provision, [22] that provide evidence about health status and health utilisation [27]. Finally, WHO Europe has provided important leadership, publishing a WHO Strategy and Action Plan (SAAP) for Refugees and Migrants in Europe. This provides a firm policy imperative for advancing the field, with guidance on nine strategic areas for priority action (Table 2) [28]. The implementation of this policy in each country requires sound and up-to-date national evidence for policy-makers and practitioners alike. The objective of this study was, therefore, to conduct a scoping review of existing peer-reviewed research on the health of migrants in the Republic of Ireland and to use the WHO-SAAP to identify any gaps in the evidence.

**Table 2:** WHO Europe strategy and action plan for refugee and migrant health: strategic areas

*1. Establishing a framework for collaborative action*	The aim is to promote and strengthen collaborative action on migrant health issues among international, national and local organisations and institutions.
*2. Advocating for the right to health of refugees*	The aim is to contribute with factual and precise information to reduce discrimination and stigmatisation, and to eliminate barriers to health care for refugees and migrants.
*3. Addressing the social determinants of health*	The aim is to build upon an adequate policy dialogue on the health of refugees, asylum seekers and migrants across all the involved government states and public.
*4. Achieving public health preparedness and ensuring an effective response*	The aim is to incorporate the health needs of refugees, asylum seekers and migrants in the outlining and advancement of public health services and policies based on Health 2020.
*5. Strengthening health systems and their resilience*	The aim is to focus on the capacity to attain an accord on the healthcare system competences required to respond to the health needs of refugees and migrants.
*6. Preventing communicable diseases*	The aim is to provide the necessary capability to focus on communicable diseases in transit and destination countries.
*7. Preventing and reducing the risks posed by non-communicable diseases*	The aim is to establish that the needs of refugees and migrants form part of the national strategy for the prevention and control of non-communicable diseases.
*8. Ensuring ethical and effective health screening and assessment*	The aim is to ensure that screening is risk-specific and evidence-based and to provide the real interests of refugees, asylum seekers and migrants and the host population.
*9. Improving health information and communication*	The aim is to provide the adequacy, standardisation and comparability of records on the health of refugees, asylum seekers and migrants, to facilitate access to health information.

*Source: Modified from WHO, Strategy and Action Plan for refugee and migrant health in the WHO European Region. 2016*

## Methods

This is a scoping review - a mapping of literature about a broad topic where many different study designs might be applicable - following the principles suggested by Arksey and O’Malley, with consideration of modifications suggested by Levac, Colquhoun and O’Brien [29, 30].

This work was presented at the 1st World Congress on Migration, Ethnicity, Race and Health 2017 [31].

The scoping review involved a six-stage framework.

### Stage 1: Identifying the research question

The scoping review framework suggests a broad and clearly articulated research question, defining concepts, target population, health outcomes, and scope, while accounting for the aim and rationale of the review [29, 30]. The research question for the review was: ‘What is the scope, main topics and gaps in evidence in the existing literature on health of migrants residing in the Republic of Ireland?’ The definition of migrant used in the study was that as defined in the Introduction, .whilst the definition of health was considered to be, not simply the absence of disease, but a state of bodily, mental and social well-being [32].

### Stage 2: Identifying relevant studies

The scoping review framework recommends searching multiple literature sources to increase comprehensiveness of the topic under study [29]. The initial plan was to include grey literature. However, as the database search progressed, there was a larger volume of peer-reviewed literature than anticipated, so we focused on peer-reviewed literature only. We chose the databases and search terms based on a recent comprehensive WHO review in the field of migrant health, [1] and consulted a medical librarian in the University of Limerick regarding its adaptation for this review. We systematically searched 11 electronic databases: Psychinfo, PsycArticles, CINAHL, Medline, Academic Search Complete, Social Sciences Full Text (H.W. Wilson), Cochrane library, Embase, Web of Science, Econlit, and Lenus. The terms below were used in this search:

(asylum* OR refugee* OR migrant* OR migrat* OR emigrant* OR emigrat* OR immigrant* OR nomad* OR foreign* OR ethnic* OR displaced OR stateless OR state-less OR noncitizen* OR non-citizen* OR outsider* OR newcomer* OR “newly arrived” OR “new arrival*” OR “recent entrant*” OR “non national” OR non-national) AND (health*) AND (Ireland* OR Irish*).

### Stage 3: Study selection

Inclusion and exclusion criteria were established through an iterative process. We agreed on the initial selection criteria based on the research question and by focusing on empirical research on the health of migrants in the Republic of Ireland. All study designs, intervention types, and migrant groups were included. The final selection criteria are shown in Additional file 1.

### Stage 4: Data charting

We used EndNote to manage retrieved items and extracted the data under the following headings: authors, publication year, title of the study, geographic location of the study, data collection period, study design, target population, target migrant group, definition of migrant group, participant group, study objective(s), data collection methods, and main study findings. We also recorded whether the study had a primary focus on migrant health or not. If authors made an explicit link between the study rationale and migration or migrant health, it was classified as having a ‘primary focus’ on migrant health. Their definition of ‘migrant’ was also recorded. If authors did not make this explicit link, the study was not classified as having a primary focus and details of data relevant to migrant health reported in the paper were recorded.

Although there are no well-established criteria for a scoping review to quality-appraise included studies, we added this step to maximise the robustness of our review. We used the criteria suggested by Kuper et al. [33] for qualitative studies; the Effective Public Health Practice Project Quality Assessment Tool (EPHPP) for cross-sectional quantitative designs, and the Newcastle Ottawa Scale [34, 35] for cohort studies. We classified the methodological quality of all studies as low, moderate, or high.

### Stage 5: Collating, summarizing and reporting results

We used the stated aims and objectives of each included paper to identify the main research topic. We then deductively analysed and categorised each paper to the nine strategic areas in the WHO’s SAAP (see Table 2), [28] by iteratively developing coding rules during the analysis process.

NV and SP completed the first round of analysis. NV, AH and AM conducted the second round of analysis using independent categorisation of papers, followed by team meetings to examine any discrepancies and to develop clear guidelines for categorisation of papers to one or more strategic areas (studies sometimes covered issues in more than one strategic area). The findings of the analysis were shared with SP and SS for critical commentary, and the final analysis was agreed by all authors.

## Results

**Fig. 1:**
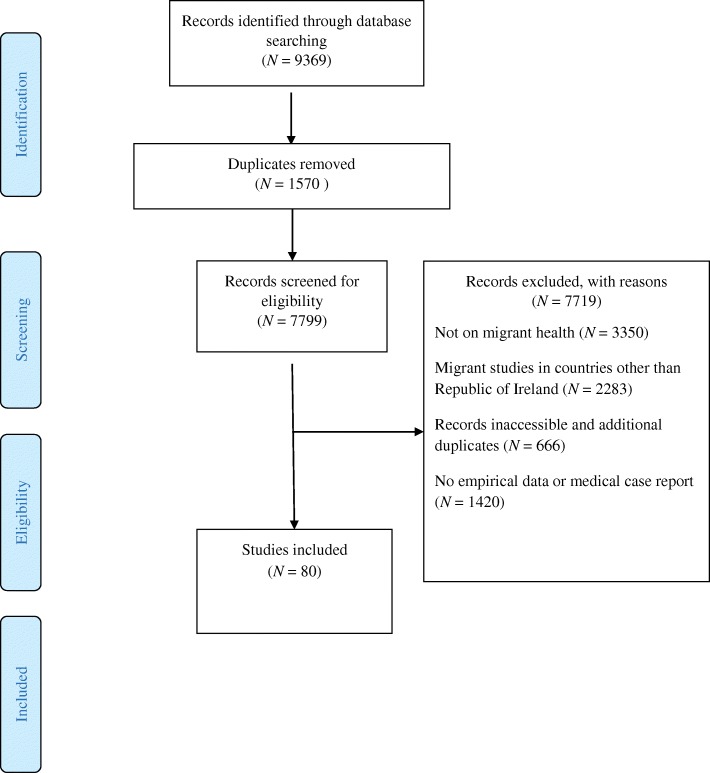
PRISMA flow chart illustrating selection of records included in scoping review

A Preferred Reporting Items for Systematic Reviews and Meta-Analyses (PRISMA) flow diagram is given in Fig. 1 and a completed PRISMA checklist is included as Additional file 2. Of 7799 records screened, 3350 (43%) were excluded because they were unrelated to migrant health and 2283 (29%) were excluded because they involved migrants in countries other than the Republic of Ireland. We identified 80 articles about migrant health in the Republic of Ireland. These are listed and numbered S1 to S80 in Additional file 3 and summarised in table form in Additional file 4.

### Overall findings

The majority of studies were published since 2009 (*n* = 65, 81%), and had a primary focus on migrant health (*n* = 66, 82%). The others had a secondary focus which were population-based papers that collected demographic data relevant to migration, such as country of birth, ethnicity or nationality.

Of the available information on funding (78/80 papers), a funding source was acknowledged for 39 (50%) of them, most commonly the Health Research Board, which is the main funding body for health research in Ireland (19 of the 39, 49%).

Of the 80 papers, 38 (48%) were academic authorship only, 22 (28%) were joint academic/healthcare or clinical academic authors, 11 (14%) were healthcare only and the remaining nine (11%) included mixtures of academic/community/advocacy/government/healthcare authorships (one had no affiliation).

A standardised definition for the migrant population of interest was provided in only 12% [S9, S30, S37, S53, S54, S55, S59, S71, S72, S78] of papers and these were mostly for refugees and asylum seekers with reference to UNHCR definitions. Fifty other studies provided a description of the population of interest in their specific study, with considerable variation in the use of terminology.

Of the included studies, 28 (35%) had a qualitative design, and 52 (65%) a quantitative design. For qualitative studies, in general the authors did not report which specific qualitative tradition or design [31] they had used, and the most common method for data collection was semi-structured interviews (*n* = 10). Quantitative studies were mainly cross-sectional (*n* = 23, 29%) and retrospective case series (*n* = 2, 3%), with three cohort studies and one case–control study. Based on the quality appraisal criteria, more than half of the articles (57%) were of low quality, 29% moderate quality, and 14% high quality. The low-quality studies were categorised as such because they were not representative of the target populations and/or did not provide enough information about the study design to complete the appraisal.

10% of the studies (*n* = 8) were based in a primary care setting. In eight studies (10%), data from Ireland were included as part of a larger international study. They related to a number of topics: 20 on maternal health [S11, S15, S18, S20,S 22, S25, S30, S32, S34, S36, S47, S49, S62, S63, S67, S71, S72, S73, S76, S79] 16 on access and utilisation of healthcare [S21, S35, S37, S38, S39, S42, S44, S46, S51, S53, S54, S65, S68, S69, S70, S74], 15 on infant and child health [S3, S6, S10, S16, S33, S40, S43, S50, S55, S57, S58, S64, S66, S75, S77], nine on infectious diseases [S1, S2, S4, S7, S19, S29, S31, S45, S52], eight on diversity in healthcare staffing [S5, S8, S9, S13, S17, S23, S24, S41], six on mental health [S28, S56, S59, S70, S78, S80] and six on ‘other’ topics such as occupational health [S12, S14, S60] and health behaviours [S26, S27, S48].

We categorised all studies onto the WHO-SAAP strategic areas to identify gaps in evidence, to which we now turn.

### Categorisation of included studies to the WHO-SAAP strategic areas

**Fig. 2:**
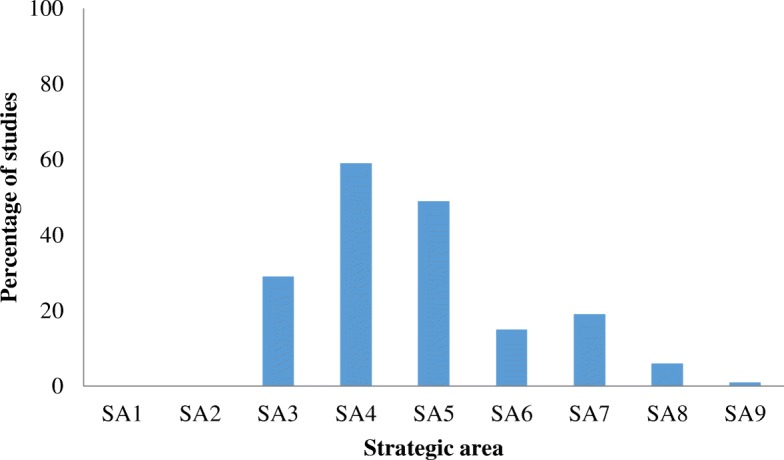
Percentage of studies categorised to each strategic area (*n* = 80)

The categorisation of included studies to the WHO-SAAP strategic areas is shown as Fig. 2. There were no studies categorised to strategic area 1, on establishing a framework for collaborative action, or strategic area 2, on advocating for the right to health of refugees, asylum seekers and migrants.

### Strategic area 3: Addressing the social determinants of health

Twenty-three studies (29%) (Additional file 1 [S11–S14, S16, S17, S21, S23, S24, S32, S48, S49, S51, S57, S59, S60, S66, S67, S70, S73, S77, S79, S80]) addressed the social determinants of health. The majority (*n* = 16, 70%) had a quantitative design and a primary focus on migrant health (*n* = 22, 96%). Employment was the focus of seven studies (30%), including the prevalence of work-related injuries in migrant workers compared to the host population [S12, S14, S60] and the experience of migrant workers in the healthcare system [S13, S17, S23, S24]. Over half of these studies (*n* = 13, 56%) were also categorised to strategic area 4 and addressed a range of issues; exploring the evidence for a ‘healthy immigrant effect’ [S48, S49]; the relationship between legal status and health outcomes [S59, S70]; and the influence of country of birth, ethnicity and citizenship on breastfeeding behaviours [S11, S32, S79].

### Strategic area 4: Achieving public health preparedness and ensuring an effective response

The majority of studies (*n* = 47, 59%) were categorised to strategic area 4 [S4, S3, S6, S11, S15, S16, S18–S20, S22, S25, S28–S34, S40, S43–S50, S53, S55–S65, S67, S70, S72, S73, S75, S76, S78–S80], mostly in the context of providing evidence on the health needs of migrants. The majority of these studies had a quantitative design (*n* = 36, 77%) and a primary focus on migrant health (*n* = 38, 80%), and were also categorised to another strategic area (*n* = 42, 89%), most commonly strategic area 5 (*n* = 19, 40%) and strategic area 3 (*n* = 13, 28%). Studies which were only categorised to strategic area 4 focused on maternal or child health [S15, S22, S25, S40, S50].

### Strategic area 5: Strengthening health systems and their resilience

Half of the studies (*n* = 39, 49%) addressed the health system’s capacity to respond to the needs of migrants and refugees [S5, S8, S9, S13, S17, S21, S23, S24, S30, S35–S39, S41–S44, S46, S47, S51, S53–S56, S58, S60–S65, S68, S71, S72, S74–S76, S78]. Migrant health was the primary focus of the majority of these studies (*n* = 36, 92%), with the remaining three studies providing information on the health system’s capacity through an analysis of findings by demographic characteristics, including country of birth [S43, S47, S75]. Of the 39 studies, 24 (62%) had a qualitative design.

Seventeen studies focused on healthcare workers (nurses, midwives, hospital-based doctors, general practitioners, primary and social care providers, carers). Nine of these studies [S21, S36, S38, S42, S53, S54, S61, S72, S74] explored the experiences of healthcare workers in providing care to migrants and refugees and their own training needs to ensure the delivery of culturally sensitive healthcare. Eight of these studies [S5, S8, S9, S13, S17, S23, S24], and [S41], focused on migrant healthcare workers in the health system, including a survey of their perceptions and attitudes to integration and their plans to remain working in Ireland.

Language barriers, the lack of access to interpreting services and the need for implementation of guidelines for effective cross-cultural communication were addressed in 12 studies, with a range of stakeholders and settings [S35, S36, S37, S38, S39, S42, S51, S55, S71, S72, S74, and S78].

Nine studies focused on maternity services, including migrants’ experiences of these services [S30, S62, S63, S71]; the experience of maternity service providers in caring for migrant and refugee women [S36, S72]; and a comparison of delivery and perinatal outcomes by country of birth [S47, S75, S76].

### Strategic area 6: Preventing communicable diseases

Twelve (15%) studies focused on communicable diseases: four on Human Immunodeficiency Virus (HIV) [S1, S3, S4, S52], four on tuberculosis [S2, S19, S29, S45], two on malaria [S7, S33], one on communicable diseases in childhood [S16] and one on infectious rashes in pregnant women [S31]. The majority (*n* = 10, 83%) did not have a primary focus on migrant health but profiled the participants and analysed findings by demographic characteristics, including country of birth. Eight studies were retrospective chart reviews in one or two hospital clinics and, while they provided some information on epidemiological surveillance, they are not population representative. One qualitative study explored the challenges and opportunities for HIV testing in African migrants [S1]. Where universal access was provided, e.g. vaccinations for childhood communicable diseases, one population-representative cohort study explored the ethnicity of the mother and uptake of vaccinations [S16].

### Strategic area 7: Preventing and reducing the risks posed by non-communicable diseases

Fifteen (19%) studies [S6, S10, S12, S14, S18, S20, S26, S27, S28, S34, S43, S47, S58, S69, and S73] addressed the risks of non-communicable diseases. All were quantitative in design and the majority (*n* = 10, 67%) had a primary focus on migrant health. Five studies focused on nutrition and alcohol use, all in pregnant women, including studies on their diet [S34], body composition [S18], weight gain [S20], alcohol consumption [S47] and Vitamin D status [S73]. Two studies focused on the prevalence of smoking; one with a primary focus on migrant health comparing Polish migrants’ smoking habits to the host population [S26]. Only two studies had an explicit aim to compare health literacy between the host population and migrants; one in the context of diabetes self-care [S69] and one on Vitamin D status in pregnancy [S73].

### Strategic area 8: Ensuring ethical and effective health screening and assessment

Five (6%) studies addressed health screening, all in the context of communicable diseases (two on HIV [S1, S52]; one on tuberculosis [S19], one on communicable diseases in childhood [S16] and one on infectious rashes in pregnant women [S31]). All five studies were also categorised under strategic area 6, and four had a quantitative design. Two of the studies had a primary focus on migrant health; one which explored the immunity to infectious rashes in pregnancy by nationality of the mother (including the host population) [S31], and one which explored the challenges and opportunities for HIV testing in African migrants [S1]. There were no examples of research on health screening and assessment for non-communicable diseases in migrants.

### Strategic area 9: Improving health information and communication

Only one study was categorised to Strategy 9. It used participatory research methods to involve migrants and other stakeholders in a dialogue to develop a guideline for effective cross-cultural communication in general practice consultations [S51]. No studies were focused on strengthening health information systems for improved data collection on migrant health.

## Discussion

### Summary of results

This is a comprehensive scoping review of peer-reviewed published research about migrant health in the Republic of Ireland. To our knowledge, it is the only review that uses the WHO-SAAP (2016) [28] as a framework to analyse the field and develop recommendations. There is a significant amount of research activity, particularly since 2009. Most research is quantitative, with a primary focus on migrant health. Few studies provided a standardised definition for the migrant population of interest, but most provided a working definition for their project. Most studies are of low–moderate quality. They tend to be regional in focus with very few examples of international collaborations. A large amount of the research is relevant to the social determinants of health, public health preparedness and health system adaptations as described in the WHO Strategy (strategic areas 3, 4 and 5 respectively) and a lesser amount is relevant to the study of communicable and non-communicable diseases and health screening (strategic areas 6, 7 and 8 respectively). There is a dearth of research about frameworks for collaborative action, advocacy and human rights and improving health information systems (strategic areas 1, 2 and 9 respectively).

### Methodological critique

We were rigorous in our approaches to all stages of the review, which adhered to the key principles of the scoping review framework [29, 30] and PRISMA guidelines. An optional stage in scoping reviews for stakeholder engagement, recommended by Levac et al. [30], was not conducted. We did however, have representation from policy (SS, SP and academic sectors (SP NV, AH, AM) in our review team. Our process included a quality appraisal, which is beyond the recommendations in the literature for scoping reviews [29, 30].

There were limitations to our search. This scoping review only includes studies within our search capacity (e.g. accessible on databases searched). The database searches did not include the grey literature because the amount of relevant peer-reviewed literature surpassed expectations. This means that there may be research on WHO priority areas that we have not identified.

Using the WHO SAAP [28] as our analytic framework was a strength as it allowed us to locate the Irish research in an international policy context, which is important for strengthening the imperative for responses by policy makers, service planners and practitioners. This is also positive for developing international research collaborations. It was analytically challenging at times to differentiate between the strategic areas, particularly because of some overlap in the description of areas 3, 4 and 5: social determinants of health and public health preparedness and health system reliance. Our analysis does not synthesise the findings per topic and per migrant group. This is the logical next step for expanding the evidence base on migrant health in Ireland, as is the conduct of systematic reviews on the evidence available around specific topics and groups.

### Connections with existing literature

This scoping review is a major contribution to the field in Ireland because it updates two previous reviews [25, 26]. It provides an apposite update on evidence about migrant health that can be used to monitor future trends in the amount of research activity being conducted and to identify relevant research questions to expand the evidence base.

The majority of studies do not provide a standardised definition of which migrant group they are concerned with, and the ones that do tend to be about refugees and asylum seekers, using the UNHCR definitions as an authoritative source. Others provide a project-specific definition. This is in keeping with findings from a recent review of definitions and terminology in the field [1].

The finding that a large number of studies focused on the social determinants of health, public health preparedness and health system adaptations (strategic areas 3, 4 and 5), with a smaller number focused on communicable diseases and health screening (strategic areas 6 and 8), contrasts with the results of a previous scoping review on migrant health in Spain, which found that the epidemiology of infectious diseases was the predominant theme [36]. Similarly, a review of research on the health of Latin-American migrants in Europe found that the largest proportion of research focused on communicable diseases (38%), with few studies addressing health determinants, including health service use (8%) and broader structural and socio-economic factors (7%) [37]. It was also interesting to note that there was a primary focus on migrant health across all the strategic areas relating to public health preparedness and health system adaptations (strategic areas 4 and 5). In contrast, studies that related to communicable diseases and screening (strategic areas 6 and 8) had a primary focus on a specific disease, such as tuberculosis, and variables examined included those that relate to migrants (for example, country of origin or ethnicity). This indicates that, even though migrants are coming from countries where the prevalence of communicable diseases is higher than in Ireland, [38] researchers in Ireland who set out to generate empirical data about migrant health to date do not appear to have directed their attention to negative and risky aspects of migrant health for the host population. These findings are in contrast with Spanish research, [36] which highlights that the research landscape on migrant health can vary considerably by country/migrant population group. This points to the WHO-SAAP as a helpful framework to elucidate the approaches to migrant health research within and across countries, as well as its role in identifying research gaps.

With regard to research gaps in the Irish setting, we note a lack of studies about collaborative action, advocacy on human rights and improving health information systems (strategic areas 1, 2 and 9). This finding warrants attention. It suggests that health researchers would benefit from thinking about action orientation studies and collaborations with scholars of law and political science. This would create valuable cross-fertilisation of ideas to generate relevant inter-disciplinary projects. On the specific issue of health information systems, this certainly needs attention. The need for a robust and comparable evidence base within and across countries, with involvement of migrants, has been well argued [1]. Some of the authors of this review are involved in a participatory research project to explore: (i) where data about health and ethnicity in Ireland are routinely collected in information systems and (ii) the implementation of an ethnic identifier in primary care [39, 40].

The nature of the evidence base, for example the dominance of quantitative studies and the low methodological quality of studies, is noted. This finding resonates with concerns which have been previously raised over the methodological quality of research in the field of migrant health [41].

We found that evidence about migrant health may be found in studies whose primary focus was not on migrant health; studies that provide information on the topic through an analysis of findings by demographic characteristics, including country of birth. This finding reflects the nature of the field which is not neatly bound, and where migrant health issues are not always different from the native population’s health issues. The inter-relationships between migrant and ethnic minority health are important and well documented; for example, the WHO published a briefing on how health systems can address health inequities for migrants and ethnic minority groups [42]. The recognised need for attention to structural and intersectional aspects of migrant health is also relevant [43].

### Implications for policy, practice and research

Findings of this review should be presented to key stakeholders in the Irish Government and Health Service Executive so that the current and evolving policy and practices around migration and integration are informed by evidence. Establishing a national Centre for Migrant Health, following international best practice in countries such as Norway, Denmark and the Former Yugoslav Republic of Macedonia, [22] would be a major step forward for policy and practice. This would be a significant mechanism for moving past current ad-hoc arrangements for co-ordinating and consolidating knowledge and its dissemination [22].

The gaps in knowledge identified using the WHO-SAAP about collaborative action, advocacy and human rights and improving health information systems need attention to expand the evidence base, and a detailed synthesis of findings about other areas should, as mentioned above, be conducted.

## Conclusion

The evidence base about migrant health in Ireland is growing considerably. The WHO-SAAP was a useful framework for analysing the nature and relevance of the research, revealing some key gaps to be addressed. This process could be replicated to scope research on migrant health in other countries. This would provide a comparable and robust oversight of the state of the art within, and across, countries.

## Additional files

Additional file 1: Table of Inclusion and Exclusion Criteria. This file provides information on inclusion and exclusion criteria used to appraise papers for the scoping review. (DOCX 13 kb)

Additional file 2: PRISMA 2009 checklist. This file shows how the scoping review meets the criteria for the PRISMA checklist. (DOC 51 kb)

Additional file 3: List of studies included in the Scoping review (S1-S80). This file provides the full reference for each paper included in the scoping review. (DOCX 24 kb)

Additional file 4: Summary Table of Included Papers. This file provides summary information on each paper included in the scoping review. There are details of the citation; whether migrant health was a primary/secondary focus of the research; authors’ description of migrant population OR information on data collected relevant to migration; study design; main research topic; and WHO SAAP Strategic Area. (DOCX 19 kb)

## Abbreviations

EEA: European Economic Area
EPHPP: Effective Public Health Practice Project Quality Assessment Tool
EU: European Union
HIV: Human Immunodeficiency Virus
IOM: International Organisation for Migration
PRISMA: Preferred Reporting Items for Systematic Reviews and Meta-Analyses
RPP: Refugee Protection Programme
SAAP: Strategy and Action Plan
UNHCR: United Nations High Commissioner for Refugees
WHA: World Health Assembly
WHO: World Health Organisation
