# Supplementary material for: Migrant health research in the Republic of Ireland: a scoping review
Source: BMC Public Health. 2019 Mar 20;19:324. doi: 10.1186/s12889-019-6651-2 (PMC6425684; doi:10.1186/s12889-019-6651-2)
Supplement: Supplementary file 1 — Table of Inclusion and Exclusion Criteria. This file provides information on inclusion and exclusion criteria used to appraise papers for the scoping review. (DOCX 13 kb) [file 12889_2019_6651_MOESM1_ESM.docx]

**Supplementary File 1: Table of Inclusion and Exclusion Criteria**

| **Inclusion and exclusion criteria** |  |
| --- | --- |
| Inclusion criteria | Articles were included if:   - Research was based on primary or secondary data on the health of migrants in the Republic of Ireland (ROI) - Peer-reviewed publications - Articles in English language - Published from January 2001 to April 2017 |
| Exclusion criteria | Articles were excluded if:   - No analysis of primary or secondary data was included, i.e. editorial, discussion articles, study protocols, methods papers, literature reviews - Medical case reports - Non-human studies: animal studies, plant studies, genetic studies, cell studies - Non-migrant health studies - Migrant studies in other countries including Northern Ireland - Research about Irish travellers* - No full text available - Studies about migrants in the ROI, but not related to their health status and not conducted in healthcare settings |

** In Ireland, Irish travellers are officially regarded as an ethnic minority since 2017.* [*http://www.paveepoint.ie*](http://www.paveepoint.ie)
