# Supplementary material for: Migrant health research in the Republic of Ireland: a scoping review
Source: BMC Public Health. 2019 Mar 20;19:324. doi: 10.1186/s12889-019-6651-2 (PMC6425684; doi:10.1186/s12889-019-6651-2)
Supplement: Supplementary file 3 — List of studies included in the Scoping review (S1-S80). This file provides the full reference for each paper included in the scoping review. (DOCX 24 kb) [file 12889_2019_6651_MOESM3_ESM.docx]

**Supplementary File 3: List of studies included in the Scoping review (S1-S80)**

- S1. Adedimeji A, Asibon A, O’Connor G, Carson R, Cowan E, McKinley P, et al. Increasing HIV testing among African immigrants in Ireland: challenges and opportunities. J Immigr Minor Health 2015;17(1):89-95.
- S2. Ajagbe OB, Kabair Z, O’Connor T. Survival analysis of adult tuberculosis disease. PLoS ONE 2014;9(11).
- S3. Al-Assaf N, Maoldomhnaigh CO, Gavin P, Butler K. Paediatric HIV: the experience in Ireland 2004–2011. Irish Med J 2013;106(7).
- S4. Babineau K, Dea SO, Courtney G, Clancy L. Smoking behaviour among people living with HIV and AIDS: a sub-group comparison. Irish Med J 2016;109(4):386.
- S5. Beagan BL, Chacala A. Culture and diversity among occupational therapists in Ireland: when the therapist is the ‘diverse’ one. Br J Occup Ther 2012;75(3):144-51.
- S6. Bolton S, McDonald D, Curtis E, Kelly S, Gallagher L. Autism in a recently arrived immigrant population. Eur J Pediatr 2014;173(3):337-43.
- S7. Breslin TM, Ionmhain UN, Bergin C, Gallagher D, Collins N, Kinsella N, et al. Malarial cases presenting to a European urban Emergency Department. Eur J Emerg Med 2013;20(2):115-9.
- S8. Brugha R, McAleese S, Dicker P, Tyrrell E, Thomas S, Normand C, et al. Passing through – reasons why migrant doctors in Ireland plan to stay, return home or migrate onwards to new destination countries. Hum Resources Health. 2016;14:45-54.
- S9. Bruyneel L, Li B, Aiken L, Lesaffre E, Van den Heede K, Sermeus W. A multi-country perspective on nurses’ tasks below their skill level: reports from domestically trained nurses and foreign trained nurses from developing countries. Int J Nurs Stud 2013;50(2):202-9.
- S10. Carroll A, Onwuneme C, McKenna MJ, Mayne PD, Molloy EJ, Murphy NP. Vitamin D status in Irish children and adolescents: value of fortification and supplementation. Clin Pediatr 2014;53(14):1345-51.
- S11. Castro PD, Layte R, Kearney J. Ethnic variation in breastfeeding and complementary feeding in the Republic of Ireland. Nutrients 2014;6(5):1832-49.
- S12. Connell PP, Saddak T, Harrison I, Kelly S, Bobart A, McGettrick P, et al. Construction-related eye injuries in Irish nationals and non-nationals: attitudes and strategies for prevention. Irish J Med Sci 2007;176(1):11-4.
- S13. Cummins T. Migrant nurses’ perceptions and attitudes of integration into the perioperative setting. J Adv Nurs 2009;65(8):1611-6.
- S14. Davidson CC, Orr DJ. Occupational injuries in foreign-national workers presenting to St James’s Hospital Plastic Surgery service. Irish Med J 2009;102(4):108-10.
- S15. Dempsey M, Peeren S. Keeping things under control: exploring migrant Eastern European women’s experiences of pregnancy in Ireland. J Reprod Infant Psyc 2016;34(4):370-82.
- S16. Doherty E, Walsh B, O’Neill C. Decomposing socioeconomic inequality in child vaccination: results from Ireland. Vaccine 2014;32(27):3438-44.
- S17. Doyle M, Timonen V. The different faces of care work: understanding the experiences of the multi-cultural care workforce. Ageing Soc 2009;29(3):337-50.
- S18. Farah N, Murphy M, Ramphul M, O’Connor N, Kennelly MM, Turner MJ. Comparison in maternal body composition between Caucasian Irish and Indian women. Journal of Obstetrics & Gynaecology. 2011;31(6):483-5.
- S19. Fitzgibbon MM, Gibbons N, Roycroft E, Jackson S, O’Donnell J, O’Flanagan D, et al. A snapshot of genetic lineages of *Mycobacterium tuberculosis* in Ireland over a two-year period, 2010 and 2011. Eurosurveillance 2013;18(3).
- S20. Heery E, Kelleher CC, Wall PG, McAuliffe FM. Prediction of gestational weight gain – a biopsychosocial model. Publ Health Nutr 2015;18(8):1488-98.
- S21. Henry A, Timmins F. An exploration of specialist palliative care nurses’ experiences of providing care to hospice inpatients from minority ethnic groups – implication for religious and spiritual care. Religions 2016;7(2).
- S22. Hughes A, Gallagher S, Hannigan A. A Cluster analysis of reported sleeping patterns of 9-month old infants and the association with maternal health: results from a population based cohort study. Matern Child Health J 2015;19(8):1881-9.
- S23. Humphries N, Brugha R, McGee H. “I won’t be staying here for long”: a qualitative study on the retention of migrant nurses in Ireland. Hum Resources Health 2009;7.
- S24. Humphries N, Tyrrell E, McAleese S, Bidwell P, Thomas S, Normand C, et al. A cycle of brain gain, waste and drain – a qualitative study of non-EU migrant doctors in Ireland. Hum Resources Health 2013;11.
- S25. Ismail KI, Marchocki Z, Brennan DJ, O’Donoghue K. Intrapartum caesarean rates differ significantly between ethnic groups—relationship to induction. Eur J Obstetr Gynecol Reprod Biol 2011;158(2):214-9.
- S26. Kabir Z, Clarke V, Keogan S, Currie LM, Zatonski W, Clancy L. Smoking characteristics of Polish immigrants in Dublin. BMC Publ Health 2008;8:428.
- S27. Kabir Z, Keogan S, Clarke V, Clancy L. Second-hand smoke exposure levels and tobacco consumption patterns among a lesbian, gay, bisexual and transgender community in Ireland. Publ Health 2013;127(5):467-72.
- S28. Kelly BD, Emechebe A, Anamdi C, Duffy R, Murphy N, Rock C. Custody, care and country of origin: demographic and diagnostic admission statistics at an inner-city adult psychiatry unit. Int J Law Psychiatr 2015;38:1-7.
- S29. Kennedy B, O’Connor B, Korn B, Gibbons N, O’Connor T, Keane J. Multi-drug resistant tuberculosis: experiences of two tertiary referral centres. Irish Med J 2011;104(6).
- S30. Kennedy P, Murphy-Lawless J. The maternity care needs of refugee and asylum seeking women in Ireland. Feminist Rev 2003(73):39.
- S31. Knowles SJ, Grundy K, Cahill I, Cafferkey MT. Susceptibility to infectious rash illness in pregnant women from diverse geographical regions. Comm Dis Publ Health 2004;7(4):344-8.
- S32. Ladewig EL, Hayes C, Browne J, Layte R, Reulbach U. The influence of ethnicity on breastfeeding rates in Ireland: a cross-sectional study. Journal of epidemiology and community health. 2014;68(4):356-62.
- S33. Leahy TR, Malikiwi A, Cafferkey M, Butler KM. Imported childhood malaria: the Dublin experience, 1999–2006. Irish J Med Sci 2009;178(3):329-32.
- S34. Lindsay KL, Gibney ER, McNulty BA, McAuliffe PM. Pregnant immigrant Nigerian women: an exploration of dietary intakes. Publ Health (Elsevier) 2014;128(7):647-53.
- S35. Lionis C, Papadakaki M, Saridaki A, Dowrick C, O’Donnell CA, Mair FS, et al. Engaging migrants and other stakeholders to improve communication in cross-cultural consultation in primary care: a theoretically informed participatory study. BMJ Open. 2016;6(7).
- S36. Lyons SM, O’Keeffe FM, Clarke AT, Staines A. Cultural diversity in the Dublin maternity services: the experiences of maternity service providers when caring for ethnic minority women. Ethnic Health 2008;13(3):261-76.
- S37. MacFarlane A, Dzebisova Z, Karapish D, Kovacevic B, Ogbebor F, Okonkwo E. Arranging and negotiating the use of informal interpreters in general practice consultations: experiences of refugees and asylum seekers in the west of Ireland. Soc Sci Med (1982). 2009;69(2):210-4.
- S38. MacFarlane A, Glynn LG, Mosinkie PI, Murphy AW. Responses to language barriers in consultations with refugees and asylum seekers: a telephone survey of Irish general practitioners. BMC Fam Pract 2008;9:68.
- S39. MacFarlane A, Singleton C, Green E. Language barriers in health and social care consultations in the community: a comparative study of responses in Ireland and England. Health policy (Amsterdam) 2009;92(2-3):203-10.
- S40. Masaud T, Dunne M, Skokauskas N. Mental health of children born to immigrant parents in Ireland: a pilot study. Community Ment Health J 2015;51(1):97-102.
- S41. Mc Gonagle C, Halloran SO, O’Reilly O. The expectations and experiences of Filipino nurses working in an intellectual disability service in the Republic of Ireland. J Learn Disabil 2004;8(4):371-81.
- S42. McCarthy J, Cassidy I, Graham MM, Tuohy D. Conversations through barriers of language and interpretation. Br J Nurs 2013;22(6):335-9.
- S43. McMahon C, Callaghan CO, O’Brien D, Smith OP. The increasing prevalence of childhood sickle-cell disease in Ireland. Irish J Med Sci 2001;170(3):183-5.
- S44. McMahon J, Murphy AW, Cantillon P, Avalos G, MacFarlane A. A survey of asylum seekers’ general practice service utilisation and morbidity patterns. Irish Med J 2007;100(5).
- S45. Mei Min S, Kelly P, Byrne C, Clancy L. Antibiotic resistant tuberculosis and bovine tuberculosis in an Irish hospital population (1991 to 2001). Irish Med J 2005;98(2):38-40.
- S46. Migge B, Gilmartin M. Migrants and healthcare: investigating patient mobility among migrants in Ireland. Health & Place 2011;17(5):1144-9.
- S47. Mullally A, Cleary BJ, Barry J, Fahey TP, Murphy DJ. Prevalence, predictors and perinatal outcomes of peri-conceptional alcohol exposure – retrospective cohort study in an urban obstetric population in Ireland. BMC Pregnancy Childb 2011;11(1):27.
- S48. Nolan A. The ‘healthy immigrant’ effect: initial evidence for Ireland. Health Econ Policy L 2012;7(3):343-62.
- S49. Nolan A, Layte R. The ‘healthy immigrant effect’: breastfeeding behaviour in Ireland. Eur J Publ Health 2015;25(4):626-31.
- S50. O’Connell A, Gavin A, Kelly C, Molcho M, Nic Gabhainn S. The mean age at menarche of Irish girls in 2006. Irish Med J 2009;102(3):76-9.
- S51. O’Reilly-de Brun M, MacFarlane A, de Brun T, Okonkwo E, Bonsenge Bokanga JS, Silva M, et al. Involving migrants in the development of guidelines for communication in cross-cultural general practice consultations: a participatory learning and action research project. BMJ Open 2015;5(9):e007092.
- S52. O’Shea D, Ebrahim M, Egli A, Redmond D, McConkey S. Late presentation of HIV despite earlier opportunities for detection, experience from an Irish tertiary referral institution. Irish J Med Sci 2013;182(3):389-94.
- S53. Pieper HO, Clerkin P, MacFarlane A. The impact of direct provision accommodation for asylum seekers on organisation and delivery of local primary care and social care services: a case study. BMC Fam Pract. 2011;12:32.
- S54. Pieper HO, MacFarlane A. “I’m worried about what I missed”: GP registrars’ views on learning needs to deliver effective healthcare to ethnically and culturally diverse patient populations. Educ Health (Abingdon) 2011;24(1):494.
- S55. Prendiville T, Williamson M, Cahill P. Access of asylum seeker children to acute paediatric services. Irish Med J 2007;100(2).
- S56. Radford K. ‘Unkind cuts’: health policy and practice versus the health and emotional well-being of asylum-seekers and refugees in Ireland. J Ethn Migr Stud 2010;36(6):899-915.
- S57. Ní Raghallaigh M. The causes of mistrust amongst asylum seekers and refugees: insights from research with unaccompanied asylum-seeking minors living in the Republic of Ireland. J Refug Stud 2014;27(1):82-100.
- S58. Roche P, Moran T, Wormald R, Geraghty M, Blayney A, O’Dwyer T, et al. Obstructive sleep apnoea – is ethnicity an independent risk factor, in adenotonsillectomy patients? Internet J Pediatr Neonatol 2010;12(2).
- S59. Ryan DA, Benson CA, Dooley BA. Psychological distress and the asylum process: a longitudinal study of forced migrants in Ireland. J Nerv Ment Dis 2008;196(1):37-45.
- S60. Saeed A, Khan I, Dunne O, Stack J, Beatty S. Ocular injury requiring hospitalisation in the South East of Ireland: 2001–2007. Injury 2010;41(1):86-91.
- S61. Sansani I. Responses by health care providers in Ireland to the experiences of women refugees who have survived gender- and ethnic-based torture. Women Stud Int Forum 2004;27(4):351-67.
- S62. Shandy DJ, Power DV. The birth of the African-Irish diaspora: pregnancy and post-natal experiences of African immigrant women in Ireland. Int Migr 2008;46(5):119-42.
- S63. Sheridan CP, Yekinni I, Oyeye G, Ogunleye K, Oluyede G, O’Sullivan K, et al. Comparing birth plan preferences among Irish and Nigerian women. Br J Midwifery. 2011;19(3):172-7.
- S64. Skokauskas N, Dunne M, Gallogly A, Clark C. Ethnic minority populations and child psychiatry services: an Irish study. Child Youth Serv Rev 2010;32(10):1242-5.
- S65. Stan S. Transnational healthcare practices of Romanian migrants in Ireland: inequalities of access and the privatisation of healthcare services in Europe. Soc Sci Med (1982) 2015;124:346-55.
- S66. Stevens GWJM, Walsh SD, Huijts T, Maes M, Madsen KR, Cavallo F, et al. An internationally comparative study of immigration and adolescent emotional and behavioral problems: effects of generation and gender. J Adolesc Health 2015;57(6):587-94.
- S67. Szafranska M, Gallagher L. Polish women’s experiences of breastfeeding in Ireland. Pract Midwife 2016;19(1):30-2.
- S68. Teunissen E, Gravenhorst K, Dowrick C, Van Weel-Baumgarten E, Van Den Driessen Mareeuw F, De Brún T, et al. Implementing guidelines and training initiatives to improve cross-cultural communication in primary care consultations: a qualitative participatory European study. Int J Equity Health 2017;16(1).
- S69. Thabit H, Shah S, Nash M, Brema I, Nolan JJ, Martin G. Globalization, immigration and diabetes self-management: an empirical study amongst immigrants with type 2 diabetes mellitus in Ireland. QJM 2009;102(10):713-20.
- S70. Toar M, O’Brien KK, Fahey T. Comparison of self-reported health & healthcare utilisation between asylum seekers and refugees: an observational study. BMC Public Health. 2009;9:214.
- S71. Tobin C, Murphy-Lawless J, Beck CT. Childbirth in exile: asylum seeking women’s experience of childbirth in Ireland. Midwifery 2014;30(7):831-8.
- S72. Tobin CL, Murphy-Lawless J. Irish midwives’ experiences of providing maternity care to non-Irish women seeking asylum. Int J Womens Health. 2014;6(1):159-69.
- S73. Toher C, Lindsay K, McKenna M, Kilbane M, Curran S, Harrington L, et al. Relationship between vitamin D knowledge and 25-hydroxyvitamin D levels amongst pregnant women. J Hum Nutr Diet 2014;27(3):261-9.
- S74. Tuohy D, McCarthy J, Cassidy I, Graham MM. Educational needs of nurses when nursing people of a different culture in Ireland. Int Nurs Rev 2008;55(2):164-70.
- S75. Unterscheider J, O’Donoghue K, Daly S, Geary MP, Kennelly MM, McAuliffe FM, et al. Fetal growth restriction and the risk of perinatal mortality – case studies from the multicentre PORTO study. BMC Pregnancy Childbirth 2014;14:63.
- S76. Walsh J, Mahony R, Armstrong F, Ryan G, O’Herlihy C, Foley M. Ethnic variation between white European women in labour outcomes in a setting in which the management of labour is standardised – a healthy migrant effect? BJOG 2011;118(6):713-8.
- S77. Walsh SD, De Clercq B, Molcho M, Harel-Fisch Y, Davison C, Madsen KR, et al. The relationship between immigrant school composition, classmate support and involvement in physical fighting and bullying among adolescent immigrants and non-immigrants in 11 countries. J Youth Adolescence 2016;45(1):1-16.
- S78. Welbel M, Matanov A, Moskalewicz J, Barros H, Canavan R, Gabor E, et al. Addiction treatment in deprived urban areas in EU countries: accessibility of care for people from socially marginalized groups. Drug Educ Prev Polic 2013;20(1):74-83.
- S79. Zhou Q, Younger KM, Kearney JM. An exploration of the knowledge and attitudes towards breastfeeding among a sample of Chinese mothers in Ireland. BMC Public Health 2010;10:722.
- S80. Ziarko M, Sęk H, Sieński M, Lewandowska K. Coping with stress among Polish immigrants. Health Psychol Rep 2014;2(1):10-8.
