# Supplementary material for: Migrant health research in the Republic of Ireland: a scoping review
Source: BMC Public Health. 2019 Mar 20;19:324. doi: 10.1186/s12889-019-6651-2 (PMC6425684; doi:10.1186/s12889-019-6651-2)
Supplement: Supplementary file 4 — Summary Table of Included Papers. This file provides summary information on each paper included in the scoping review. There are details of the citation; whether migrant health was a primary/secondary focus of the research; authors’ description of migrant population OR information on data collected relevant to migration; study design; main research topic; and WHO SAAP Strategic Area. (DOCX 19 kb) [file 12889_2019_6651_MOESM4_ESM.docx]

**Supplementary File 4: Summary Table of Included Papers**

| **Item ID*** | **Citation** | **Primary Focus**  **Yes /No** | **Authors’ description of migrant population**  **OR**  **Information on data collected relevant to migration** | **Study design** | **Main research topic** | **WHO-SAAP Strategic Area** |
| --- | --- | --- | --- | --- | --- | --- |
| 1. S | Adedimeji, A et al. | Yes | Black African immigrants | Qualitative | Infectious diseases | 6,8 |
|  | Ajagbe, O. B. et al. | No | Data on country of birth, race or ethnicity, and immigration status (refugee/asylum seeker) | Quantitative | Infectious diseases | 6 |
|  | Al-Assaf, N. et al. | Yes | Immigrants | Quantitative | Infant and child health | 4,6 |
|  | Babineau, K. et al. | No | Data on ethnicity & country of birth. | Quantitative | Infectious diseases | 6 |
|  | Beagan, B. L. et al. | Yes | Self-identified non-Irish ethnicity | Qualitative | Diversity in Healthcare staffing | 5 |
|  | Bolton, S. et al, | Yes | Immigrant population | Quantitative | Infant and child health | 7,4 |
|  | Breslin, T.M et al. | Yes | Immigrants from Africa | Quantitative | Infectious diseases | 6 |
|  | Brugha,R et al, | Yes | Migrant doctors | Quantitative | Diversity in Healthcare staffing | 5 |
|  | Bruyneel, L et al, | Yes | Foreign trained nurses | Quantitative | Diversity in Healthcare staffing | 5 |
|  | Carroll, A et al. | No | Data on parents’ ethnicity and country of origin | Quantitative | Infant and child health | 7 |
|  | Castro, P. D. et al. | Yes | Ethnically diverse mothers | Quantitative | Maternal health | 3,4 |
|  | Connell, P P et al, | Yes | Irish non-nationals | Quantitative | Other | 3,7 |
|  | Cummins, T | Yes | Migrant nurses | Quantitative | Diversity in Healthcare staffing | 3,5 |
|  | Davidson, C C et al. | Yes | Foreign-National Workers | Quantitative | Other | 3,7 |
|  | Dempsey, M et al, | Yes | Migrant Eastern European women – economic migrants | Qualitative | Maternal health | 4 |
|  | Doherty, E et al, | No | Data on mothers’ ethnicity | Quantitative | Infant and child health | 3,4,6,8 |
|  | Doyle, M et al, | Yes | Migrant care workers | Qualitative | Diversity in Healthcare staffing | 3,5 |
|  | Farah, N. et al, | Yes | Indian women | Quantitative | Maternal health | 4,7 |
|  | Fitzgibbon, M. M. | No | Data on global lineages | Quantitative | Infectious diseases | 4,6,8 |
|  | Heery, E et al, | No | Data on nationality | Quantitative | Maternal health | 4,7 |
|  | Henry, A. et al, | Yes | Immigrants and Minority Ethnic Groups | Qualitative | Access and utilisation of healthcare | 3,5 |
|  | Hughes, A. | No | Data on ethnic background | Quantitative | Maternal health | 4 |
|  | Humphries, N. et al, | Yes | Migrant nurses | Qualitative | Diversity in Healthcare staffing | 3,5 |
|  | Humphries, N. et al, | Yes | Non-EU migrant doctors | Qualitative | Diversity in Healthcare staffing | 3,5 |
|  | Ismail, K et al, | Yes | Eastern European women | Quantitative | Maternal health | 4 |
|  | Kabir, Z. et al, | Yes | Polish immigrants | Quantitative | Other | 7 |
|  | Kabir, Z. et al, | No | Data on Nationality | Quantitative | Other | 7 |
|  | Kelly, B. D. et al, | Yes | Individuals born outside of Ireland | Quantitative | Mental health | 4,7 |
|  | Kennedy, B. et al, | Yes | Immigrants | Quantitative | Infectious diseases | 4,6 |
|  | Kennedy, P et al, | Yes | Refugee and Asylum Seeking Women | Qualitative | Maternal health | 4,5 |
|  | Knowles, S J et al, | Yes | Immigrants | Quantitative | Infectious diseases | 4,6,8 |
|  | Ladewig, E. L. et al, | Yes | Non-Irish mothers | Quantitative | Maternal health | 3,4 |
|  | Leahy, T. R. et al, | Yes | Immigrants | Quantitative | Infant and child health | 4,6 |
|  | Lindsay, K. L. et al, | Yes | Immigrant Nigerian women | Quantitative | Maternal health | 4,7 |
|  | Lionis, C. et al, | Yes | Migrants | Qualitative | Access and utilisation of healthcare | 5 |
|  | Lyons, S. M. et al, | Yes | Ethnic minority women | Qualitative | Maternal health | 5 |
|  | MacFarlane, A et al, | Yes | Refugees and asylum seekers | Qualitative | Access and utilisation of healthcare | 5 |
|  | MacFarlane, A et al, | Yes | Refugees and asylum seekers | Quantitative | Access and utilisation of healthcare | 5 |
|  | MacFarlane, A et al, | Yes | Refugees and asylum seekers | Qualitative | Access and utilisation of healthcare | 5 |
|  | Masaud, T., et al. | Yes | Children born to immigrant parents in Ireland | Quantitative | Infant and child health | 4 |
|  | Mc Gonagle, C., et al | Yes | Filipino nurses | Qualitative | Diversity in Healthcare staffing | 5 |
|  | McCarthy, J et al, | Yes | Non Irish nationals | Qualitative | Access and utilisation of healthcare | 5 |
|  | McMahon, C. et al, | No | Data on country of origin | Quantitative | Infant and child health | 4,5,7 |
|  | McMahon, J. et al, | Yes | Asylum seekers | Quantitative | Access and utilisation of healthcare | 4,5 |
|  | Mei Min, S. et al, | No | Data on country of origin | Quantitative | Infectious diseases | 4,6 |
|  | Migge, B et al, | Yes | Migrants | Qualitative | Access and utilisation of healthcare | 4,5 |
|  | Mullally, A et al, | No | Data on nationality | Quantitative | Maternal health | 4,5,7 |
|  | Nolan A, et al, | Yes | Immigrants | Quantitative | Other | 3,4 |
|  | Nolan A, et al, | Yes | Immigrants | Quantitative | Maternal health | 3,4 |
|  | O’Connell, A et al, | No | Data on country of birth | Quantitative | Infant and child health | 4 |
|  | O’Reilly-de Brun, M. et al, | Yes | Migrants | Qualitative | Access and utilisation of healthcare | 3,5,9 |
|  | O’Shea, D et al, | No | Data on country of origin | Quantitative | Infectious diseases | 6,8 |
|  | Pieper, H et al, | Yes | Asylum seekers | Qualitative | Access and utilisation of healthcare | 4,5 |
|  | Pieper, H et al, | Yes | Ethnically and  Culturally Diverse Patient Populations | Qualitative | Access and utilisation of healthcare | 5 |
|  | Prendiville, T.et al, | Yes | Asylum seeker children | Quantitative | Infant and child health | 4,5 |
|  | Radford, K et al, | Yes | Asylum seekers and refugees | Qualitative | Mental health | 4,5 |
|  | Ní Raghallaigh, M. | Yes | Asylum Seekers and Refugees | Qualitative | Infant and child health | 3, 4 |
|  | Roche, P. et al | Yes | Immigrants | Quantitative | Infant and child health | 4,5,7 |
|  | Ryan, D et al, | Yes | Forced migrants (refugees and asylum seekers) | Quantitative | Mental health | 3,4 |
|  | Saeed, A et al, | Yes | Migrant workers from EU accession states | Quantitative | Other | 3,4,5 |
|  | Sansani, I | Yes | Refugees | Qualitative | Mental health | 4,5 |
|  | Shandy, D et al | Yes | African Immigrant Women | Qualitative | Maternal health | 4,5 |
|  | Sheridan, C. P., et al. | Yes | Nigerian pregnant women. | Quantitative | Maternal health | 4,5 |
|  | Skokauskas, N. et al, | Yes | Immigrants (first and second generations) | Quantitative | Infant and Child Health | 4,5 |
|  | Stan, S. | Yes | Romanian migrants | Qualitative | Access and utilisation of healthcare | 4,5 |
|  | Stevens, G et al, | Yes | Immigrants | Quantitative | Infant and child health | 3 |
|  | Szafranska, M. et al, | Yes | Polish women | Qualitative | Maternal health | 3,4 |
|  | Teunissen, E. et al | Yes | Migrants | Qualitative | Access and utilisation of healthcare | 5 |
|  | Thabit, H et al, | Yes | Non-Irish-national  patients | Quantitative | Access and utilisation of healthcare | 7 |
|  | Toar, M et al, | Yes | Refugees and asylum seekers | Quantitative | Access and utilisation of healthcare | 4,3 |
|  | Tobin, C et al, | Yes | Asylum seeking women | Qualitative | Maternal health | 5 |
|  | Tobin, C et al | Yes | Asylum seeker | Qualitative | Maternal health | 4,5 |
|  | Toher, C et al, | Yes | Women of Asian, Sub-Saharan African and Middle Eastern and North African origin | Quantitative | Maternal health | 3,4,7 |
|  | Tuohy, D et al, | Yes | EU citizens, non-EU citizens who  may be residents (temporary/permanent), asylum seekers and/or  refugees. | Qualitative | Access and utilisation of healthcare | 5 |
|  | Unterscheider, J et al, | No | Data on mothers’ ethnicity | Quantitative | Infant and child health | 4,5 |
|  | Walsh, J. et al | Yes | Migrants | Quantitative | Maternal health | 4,5 |
|  | Walsh, S at al, | Yes | Immigrants | Quantitative | Infant and child health | 3 |
|  | Welbel, M et al, | Yes | Asylum seekers, refugees, Irregular migrants | Quantitative | Mental health | 4,5 |
|  | Zhou, Q et al, | Yes | Chinese mothers | Quantitative | Maternal health | 3,4 |
|  | Ziarko, M et al, | Yes | Polish immigrants | Quantitative | Mental health | 3,4 |

*Source: Own elaboration.*
